# Long-acting antibody ligand mimetics for HER4-selective agonism

**DOI:** 10.1038/s41598-020-74176-9

**Published:** 2020-10-14

**Authors:** Lu Shan, Kimberly M. Cook, Nantaporn Haskins, Bilal Omar, Yu Jiang, Andrew Garcia, Adem Koksal, Vaheh Oganesyan, Kim Rosenthal, Herren Wu, William F. Dall’Acqua, Melissa M. Damschroder

**Affiliations:** 1 Antibody Discovery and Protein Engineering, AstraZeneca, Gaithersburg, USA; 2 Early Oncology, AstraZeneca, Gaithersburg, USA; 3 Clinical Pharmacology and Safety Science, R&D, AstraZeneca, Gaithersburg, USA; 4 Present Address: Biotherapeutics, Denali Therapeutics, 161 Oyster Point Blvd, South San Francisco, CA 94070 USA; 5 Present Address: Antibody Discovery and Protein Engineering, NextCure, 9000 Virginia Manor Road, Suite 200, Beltsville, MD 20705 USA

**Keywords:** Biochemistry, Biotechnology, Drug discovery, Molecular biology, Structural biology

## Abstract

Neuregulin protein 1 (NRG1) is a large (> 60–amino-acid) natural peptide ligand for the ErbB protein family members HER3 and HER4. We developed an agonistic antibody modality, termed antibody ligand mimetics (ALM), by incorporating complex ligand agonists such as NRG1 into an antibody scaffold. We optimized the linker and ligand length to achieve native ligand activity in HEK293 cells and cardiomyocytes derived from induced pluripotent stem cells (iPSCs) and used a monomeric Fc-ligand fusion platform to steer the ligand specificity toward HER4-dominant agonism. With the help of selectivity engineering, these enhanced ALM molecules can provide an antibody scaffold with increased receptor specificity and the potential to greatly improve the pharmacokinetics, stability, and downstream developability profiles from the natural ligand approach. This ligand mimetic design and optimization approach can be expanded to apply to other cardiovascular disease targets and emerging therapeutic areas, providing differentiated drug molecules with increased specificity and extended half-life.

## Introduction

Monoclonal antibodies represent the fastest-growing class of biologics, with a rapid rate of six to nine first marketing approvals per year^1^. One of the main reasons for the increased focus on antibody drug discovery is the unique target specificity and prolonged serum half-life of these agents, which provide much-needed therapeutic opportunities to patients. Antibody agonists are a highly desirable biologic drug format, with the potential to overcome the limitations of fast clearance rates and lack of receptor selectivity for a wide range of endocrines, cytokines, chemokines, and other natural ligands^2^. For receptors that require complex ligand engagement and activation, the discovery of agonistic antibodies from display libraries or immune repertoires can be hampered by the size and structural limitations of human antibodies. At the same time, this challenge provides an important opportunity to advance novel protein designs and engineering platforms to expand the potential applications of agonistic antibodies. One such example is the activation of the human epidermal growth factor receptor family member 4 (HER4) and its natural peptide agonist, neuregulin.

Neuregulins, also known as heregulins or *neu* differentiation factors, are a family of ligands that bind with low affinity to HER3 and HER4, members of the ErbB family of receptor tyrosine kinases, to activate their downstream phosphorylation and signaling. The isoform neuregulin 1 (NRG1) and its activation of the HER2/HER4 pathway is implicated in cardiomyocyte proliferation and regeneration^3–11^. The epidermal growth factor (EGF) domain in neuregulin is both necessary and sufficient for receptor binding and signaling and is being explored in a phase 3 trial as a treatment for heart failure^9,12^. The relatively large (> 60–amino-acid) peptide has a highly structured conformation consisting of two rotated β-sheets stabilized by three pairs of disulfide bonds and extensive hydrogen bonding^13–15^. Two isoforms for NRG1, NRG1α and NRG1β, are identical in sequence in the first 45 amino acids, which have been found to be the core structural sequence, whereas residues 50–63 are highly disordered and contribute little to binding affinity^16^. In crystal structures of the ErbB4/HER4 extracellular domain complexed with neuregulin-1β (NRG1b), it was revealed that NRG1b mediates an extensive conformational conversion of the receptor through extensive surface contacts to bring together two domains that are otherwise far apart in the inactive form, in a process similar to the interaction of EGF with its receptor^14,17^. By providing extensive domain contacts, neuregulin induces functional activation of both HER3 and HER4, enabling their subsequent dimerization with HER2. To explore the benefit of the HER2/HER4 pathway for cardiomyocyte functions, a more selective agonist is desired to circumvent the wider tissue expression of HER3 and avoid the neuregulin-HER3 axis in tumor biology^18,19^. HER3 and HER4 share approximately 65% sequence identity but a high degree of structural homology. In earlier efforts to improve affinity in the neuregulin peptide with phage-displayed alanine scanning and block mutation libraries, it was observed that mutants selected for enhanced HER3 binding also commuted the binding benefit to HER4^20,21^.

The structure-dependent agonistic function of NRG1b presents an interesting challenge for developing complex agonist molecules with antibody-like properties such as longer serum half-life and target selectivity. Here we describe a comprehensive strategy to build agonist antibodies with enhanced ligand selectivity. Natural neuregulin ligand was first incorporated into the complementarity-determining region 3 (CDR3) of a human antibody to generate active, fully human “antibody ligand mimetic” (ALM) molecules with optimized GGGGS linkers. We then applied a monomeric Fc (MFc) fusion technology to carry out a selectivity engineering campaign to identify neuregulin variants with enhanced HER4 selectivity and diminished HER3 binding^22^. By combining these protein engineering approaches, we were able to design ALM molecules with enhanced ligand specificity and potential to greatly improve the pharmacokinetics (PK), stability, and downstream developability profiles when compared with the natural ligand.

## Results

### ALM molecular design and optimization for active receptor signaling

**Figure 1:**
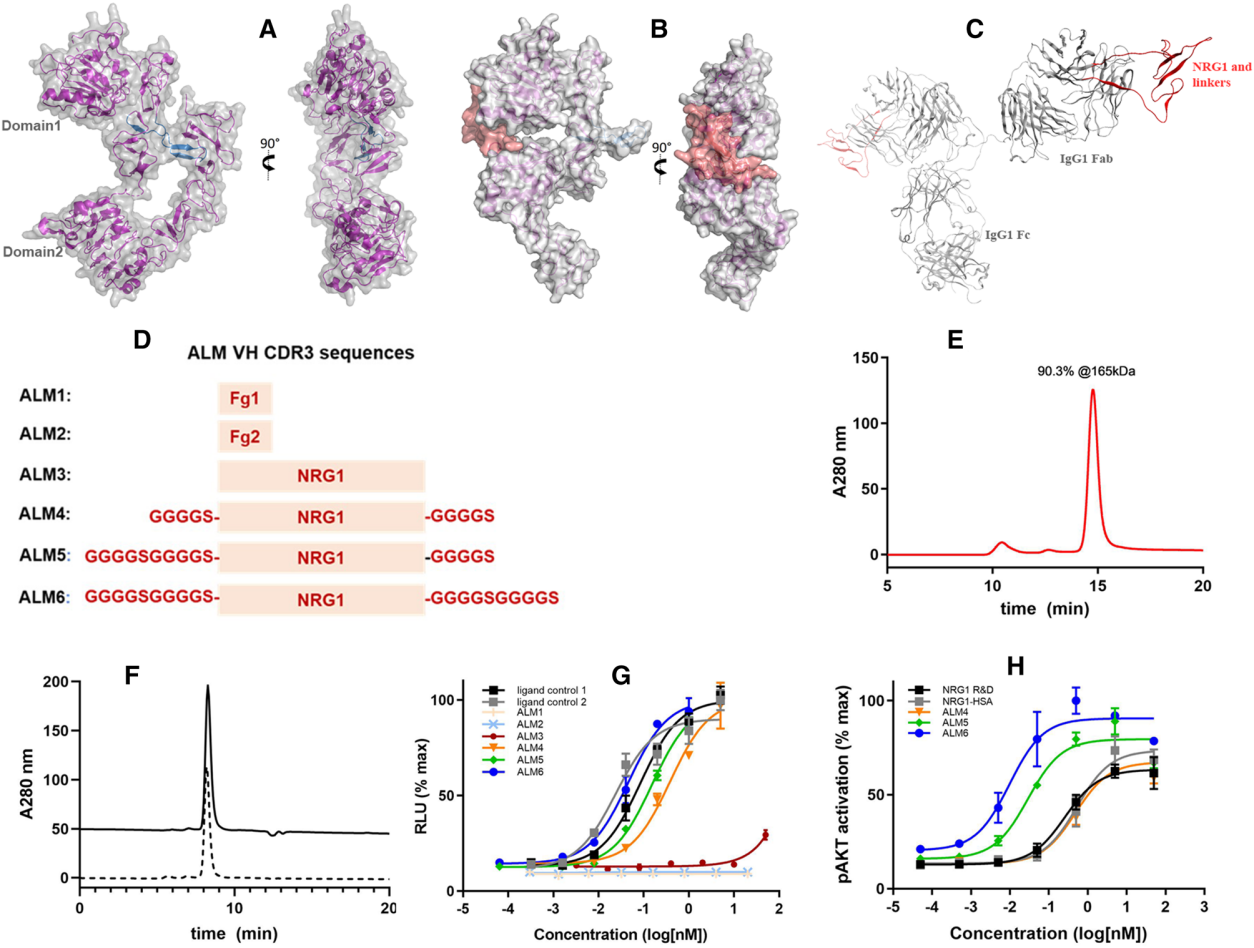
ALM design and characterization. (**A**) Unliganded structure of HER4 receptor ECD (PDB ID: 2AHX) shows the separation between domain 1 and domain 2 and a buried dimerization arm (blue). (**B**) Structure of neuregulin-bound HER4 (PBD ID: 3U7U) shows that NRG1 engage a receptor conformational change to form a binding interface with domains 1 and 2. Vertical 90-degree rotation shows the extended dimerization arm (blue). (**C**) Structural model of ALM designs, using the b12 antibody as the scaffold (PBD ID: 1HZH) and NRG1 (PDB ID: 3U7U). (**D**) The variable heavy-chain CDR3 loop was replaced with ligand sequences. (**E**) SEC-MALS analysis confirmed homogeneity of ALM6 after protein A chromatography purification, with the molecular size calculated from MALS. (**F**) SEC traces of ALM6 at 0.5 mg/mL (solid) and at 10 mg/mL (dashed). (**G**) HER2/HER4 dual-expressing HEK293 cell-based luciferase reporter assay showed a gradual increase in receptor activation from ALM3, ALM4, ALM5, and ALM6. (**H**) Phospho-AKT activation was measured on iPSC-derived cardiomyocytes. The **PyMOL** Molecular Graphics System, Version 2.4.0, Schrödinger, LLC. (https://pymol.org) was used to create the structural model images in (**A**–**C**).

The neuregulin EGF domain is a large, complex peptide consisting of 60 amino acids. The ligand conformation is sustained with three pairs of disulfide bonds and extensive hydrogen bonds, which can bind to the ECD of either HER4 or HER3 and convert the receptors from the unbound state through a major conformational change to the liganded state (Fig. 1A,B). The conformational fit by neuregulin between domains 1 and 3 of the receptor stabilizes an active form that extends the dimerization arm for subsequent homodimerization or heterodimerization with HER2 to enable downstream signaling. There have been challenges with identifying antibody agonists from phage libraries and immune repertoires (data not shown), consistent with the observation that the tertiary structure of the neuregulin ECD appears to be important for receptor activation. We henceforth envisioned a novel approach to utilize an antibody scaffold, termed “antibody ligand mimetic” (ALM), to build rationally designed antibody agonists with improved function of complex natural ligands.

Antibody CDRs are highly variable and, in the bovine antibody repertoire, are known to accommodate long peptides in the CDR3H of up to 69 amino acids in length with an extension stalk^23^. Several studies have applied the bovine “stalk-knob” architecture or a coiled-coil stalk to incorporate erythropoietin, granulocyte colony-stimulating factor, or glucagon-like peptide 1^18,24,25^. For our ALM designs, we selected the anti-HIV antibody b12 as the antibody scaffold, as it has a well-known immunoglobulin IgG1 crystal structure (Protein Data Bank [PDB]: 1HZH) and is not cross-reactive with any human proteins^26^. We used a loop grafting strategy to insert a panel of ligand peptides from the neuregulin ECD in place of the CDR3H of b12. The first two ALM molecules, ALM1 and ALM2, were designed with a 15–amino-acid peptide fragment, NEFTGDRGQNYVMAS, at the receptor domain interface in both forward and reverse directions (denoted “Fg1” and “Fg2”, in Fig. 1D). We also grafted various lengths of G4S linkers tethering the NRG1 EGF domain to the b12 CDR3H (ALM3–ALM6; Fig. 1C,D). These engineered ALM molecules were expressed in HEK293 cells; ALM1 and ALM2 were expressed at titers of > 300 mg/L, and the whole NRG1 domain–grafted ALMs had reduced expression levels, of approximately 40–150 mg/L. After purification with protein A chromatography, all the ALMs demonstrated good homogeneity, showing over 90% monomer content by size exclusion chromatography with multi-angle light scattering (SEC-MALS) (Fig. 1E).

To evaluate whether the ALMs we designed could confer HER4 binding, we used biolayer interferometry to compare the binding affinities of these ALM molecules to recombinant HER4 ECD (Table 1). We found that ALM1 and ALM2, which contained peptide fragments from the neuregulin structure, did not yield any binding with HER4. As we grafted the whole NRG1 EGF domain into the CDR3H of our scaffold antibody, even without any linkers, as seen in ALM3, we observed noticeable but very weak binding to HER4. With a stepwise increase of the G4S linker length on both the N- and the C-terminal sides of the ligand, the ALM molecules demonstrated a steady increase in HER4 binding affinity. ALM5, which has an N-terminal (G4S)2 linker and a C-terminal G4S linker, reached a binding affinity of 560 nM, similar to that of the control protein NRG1 fused with human serum albumin (HSA). Further fine-tuning of the linkers by extension of the C-terminal linker to (G4S)2 for ALM6 yielded a slightly better binding affinity, of 440 nM.

**Table 1:** Equilibrium binding of NRG1 ALM designs.

Constructs	HER4 binding K_D_
NRG1-HSA	460 nM
ALM1	NB
ALM2	NB
ALM3	> 10 μM
ALM4	5.6 μM
ALM5	560 nM
ALM6	440 nM
NRG1-MFc	640 nM

K_D_ = equilibrium dissociation constant; *NB* no binding.

The downstream signaling triggered by HER2/HER4 or HER4/HER4 receptor dimerization is known to involve the phosphorylation of ERK or AKT. To evaluate whether the designed ALM molecules exhibited agonistic activity to activate HER4-mediated dimerization and signaling, we tested these molecules in a HER2/HER4 dual-expressing serum response element (SRE)–luciferase reporter gene assay. Both HER2 and HER4 were overexpressed on HEK293 cells so that signaling through ligand-induced HER4 activation and dimerization would be detected by luciferase activity. As in the binding analysis to recombinant HER4, we observed that ALM1 and ALM2 did not show any activity, whereas ALM3, the construct without any G4S linkers, began to show marginal activity at the highest concentration (Fig. 1F). The activity of the ALM molecules increased in a stepwise fashion as the G4S linker was lengthened on the N- and C-terminal sides of the ligand, and ALM6 elicited the highest luciferase activity.

In many cell types, including cardiomyocytes, neuregulin agonism leads to activation of the phosphoinositol-3-kinase–AKT pathway, which appears to play a critical role in protecting cardiomyocytes from apoptosis^6,27^. We used cardiomyocytes derived from induced pluripotent stem cells (iPSC) to detect the activation of phospho-AKT by ALM molecules. Here we also observed a stepwise improvement of phospho-AKT activation as we increased the length of the G4S linkers in the designed constructs. Noticeably, we observed that ALM6 had a much lower EC_50_ than the NRG1-HSA control, indicating that bivalent ligand mimetics confer higher agonistic activity. These results demonstrate that with the incorporation of flexible linkers, ALMs can be designed to preserve the structural integrity of a complex ligand like neuregulin and may even have activity superior to natural ligands.

### Ligand-MFc fusion protein design and characterization

The generation of active ALM molecules that exhibit the activity of neuregulin is an important first step toward developing an agonistic antibody ligand mimetic with properties such as activity and specificity of a monoclonal antibody drug. The high degree of structural homology between HER3 and HER4 results in a similar binding pocket for NRG1 and has hampered past attempts to engineer NRG1 selectivity. Studies with phage libraries containing random block mutations have previously found that mutations that enhance HER3 binding also improve HER4 binding^20^. In a study using parallel phage selection with alanine-scanning mutagenesis, it was found that most of the residues in neuregulin were important for binding equally to both receptors^21^. In that study, when the phage hits were converted to bacteria-expressed mutants then refolded to form soluble proteins, many of the alanine variants caused loss of function to both receptors. However, seven mutants lost two-fold binding affinity to HER3 compared with HER4. Among them, mutating the NH2-terminus residues His2 and Leu3 to alanine resulted in the greatest loss of function to both receptors, but the effect was more pronounced for HER3. These data suggested that mutagenesis on neuregulin could disrupt HER3 binding more extensively compared to HER4, and it may be possible to engineer HER4-specific ligand variants.

**Figure 2:**
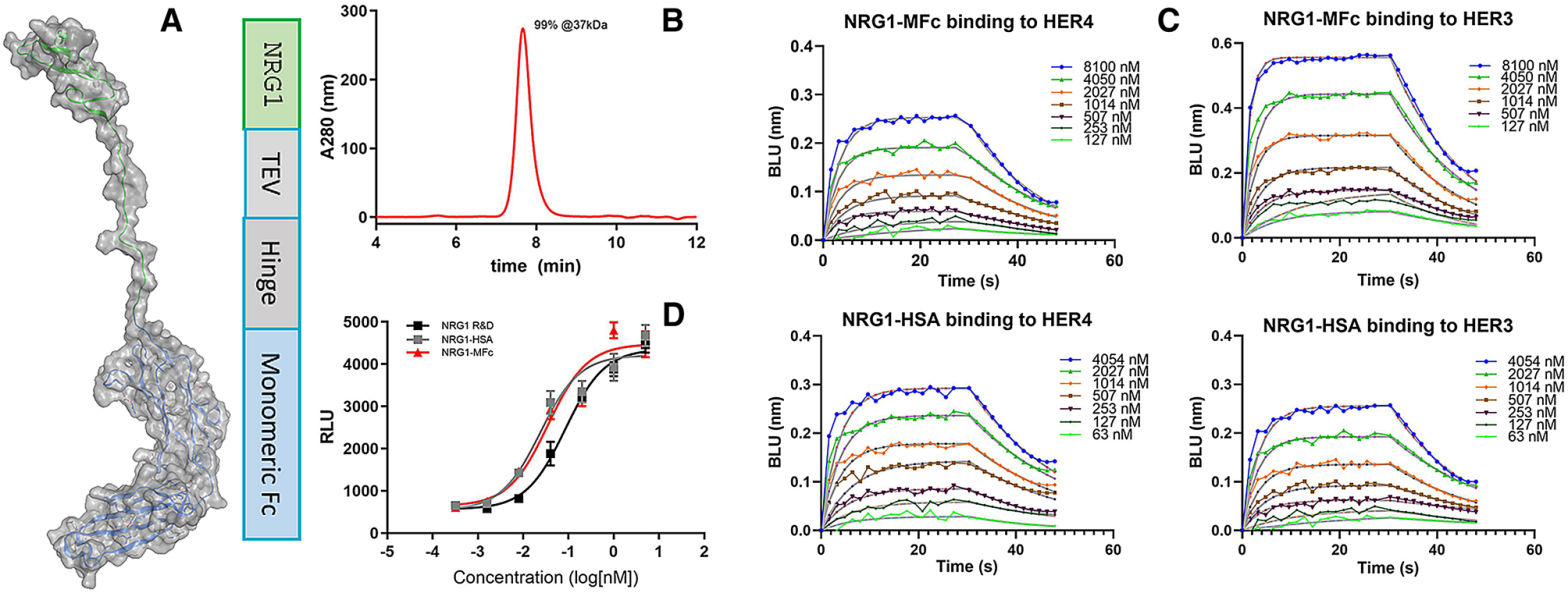
Construction of neuregulin-MFc fusion protein as an active HER4 agonist. (**A**) Structural model of the NRG1-MFc fusion protein based on the crystal structures of NRG1 (PDB ID: 3U7U) and MFc C4n variant (PDB ID: 5HVW). A TEV cleavage site and the MFc hinge region are also shown. (**B**) SEC analysis of NRG1-MFc after protein A purification showed a highly homogeneous monomeric formation, with molecular size calculated by MALS. (**C**) Octet measurements showed that NRG1-MFc bound to both HER4 and HER3, similarly to control protein NRG1 fused with HSA. (**D**) In a HER2/HER4 dual-expressing HEK293 cell-based luciferase reporter assay, NRG1-MFc activity was comparable to that of control proteins, NRG1 peptide, and NRG1-HSA. RLU = relative luminescence units. The **PyMOL** Molecular Graphics System, Version 2.4.0, Schrödinger, LLC (https://pymol.org) was used to create the structural model images in (**A**).

To engineer receptor selectivity, we set out to build an expression platform to both mimic native ligand activity and allow for a high-throughput selection strategy to fine-tune the ligand binding differential between HER4 and HER3. We designed a monovalent neuregulin fusion protein by genetically fusing the NRG1 EGF domain with a previously reported monomeric Fc domain (MFc)^22^. separated by a TEV protease cleavage site and a monomeric IgG4 hinge (Fig. 2A). We reasoned that this monomeric fusion construction would allow for mammalian expression and proper disulfide formation and protein folding while enabling a high-throughput screening platform for affinity dialing without masking avidity effects. NRG1-MFc was expressed by transient transfection in HEK293 cells, yielding a titer of approximately 30 mg/L. Single-step purification by protein A chromatography produced a homogeneous monomer of approximately 37 kDa as determined by SEC-MALS analysis (Fig. 2B). To determine whether this fusion protein exhibited native binding and activity, we used a commercially available synthetic NRG1 peptide and an NRG1-HSA fusion protein as controls. Using biolayer interferometry for direct binding to streptavidin-captured biotinylated recombinant HER3 and HER4 ECD proteins, we found that both NRG1-MFc and NRG1-HSA bound to HER4 with similar equilibrium binding affinities, of 640 and 460 nM, respectively (Table 1). As expected, NRG1-MFc bound in a similar fashion to HER4 and HER3 (Fig. 2C). Using the HER2/HER4 dual-expressing SRE–luciferase reporter assay, we showed that NRG1-MFc activated downstream signaling with a similar EC_50_ to those of NRG1-HSA and NRG1 peptide (Fig. 2D). These results demonstrated that the ligand-MFc fusion format served as an active ligand template that is suitable for ligand selectivity engineering to complement the development of an antibody agonist.

### Selectivity engineering with ligand-MFc fusion

**Figure 3:**
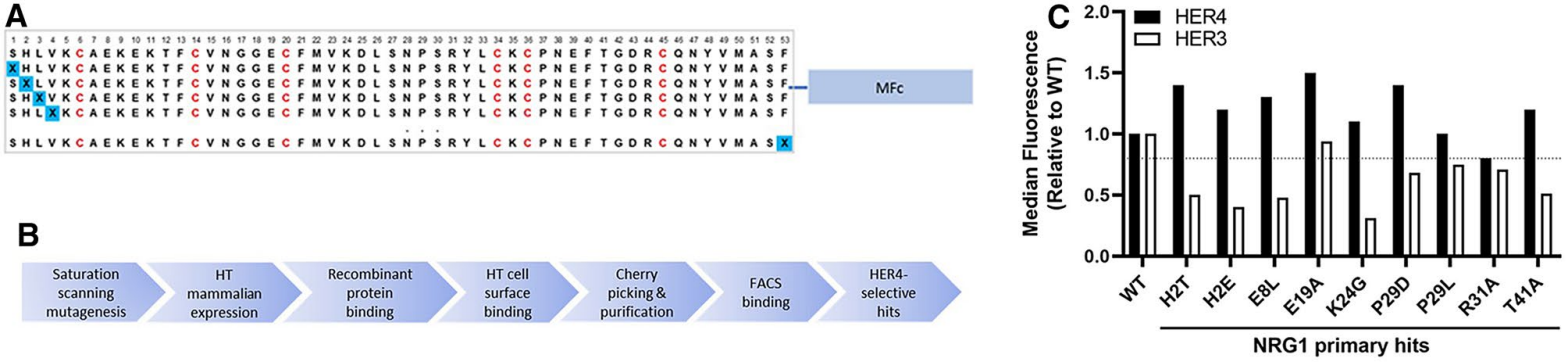
Selectivity engineering workflow design and primary screening output. (**A**) Saturation scanning mutagenesis libraries were generated for every non-cysteine residue in the template of NRG1-MFc, with a library diversity of ~ 10^3^. (**B**) Library clones were expressed at four times library size, using high-throughput mammalian transfection and expression. The supernatants were used for recombinant protein binding and HER3- and HER4- overexpressing HEK293 cell surface binding in no-wash imaging assays. The selected potential hits were re-expressed and purified. Hits that retained HER4 binding with reduced HER3 binding were determined with flow cytometry. (**C**) Primary hits were selected based on a median binding fluorescence for HER3 at a ratio of ≤ 0.8 to the parental clone and a median binding fluorescence for HER4 at or above the parental clone. WT = wild type.

The design and validation of the ligand-MFc fusion protein and the ALM antibody agonist platforms provided us with the versatility to further engineer the ligand for optimal functionality. Our aim was to preserve the HER4 agonism of the neuregulin ligand while driving the maximal reduction from HER3 binding. We designed saturation scanning mutagenesis libraries to target each of the non-cysteine residues in the NRG1 EGF domain, all of which were individually substituted with all 20 amino acids, using the NNS codon (Fig. 3A). These libraries were applied in a high-throughput selection and screening workflow (Fig. 3B). The mutation libraries were pooled before being cloned into the MFc fusion vector. More than 4,000 clones were expressed by high-throughput mammalian expression. The supernatants were harvested and screened with both enzyme-linked immunosorbent assay (ELISA) binding for recombinant HER4 and HER3, as well as a high-throughput binding assay using the Mirrorball system to HER4- and HER3-overexpressing cell lines. Clones that retained HER4 binding with reduced HER3 binding compared with the wild-type NRG1-MFc were selected and purified for confirmation by flow cytometry analysis. Most of the variants showed similar change in HER3 and HER4 binding. However, several mutations, such as the His2 to Glu mutation, showed very small but noticeable changes in their cell-based binding patterns to HER4 compared with HER3 (Fig. 3C). The primary hits from this library set were selected based on a median binding fluorescence for HER3 at a ratio of ≤ 0.8 to the parental clone and a median binding fluorescence for HER4 at or above the parental clone (Fig. 3C). A few variants containing H2T, H2E, E8L, K24G, P29D, P29L, and T41A fit the cutoff criteria. To increase the diversity coverage of the combinatorial mutagenesis design, we also included R31A because it showed greater reduction of HER3 binding, even though it bound to HER4 at a reduced level compared to the wild type.

It was unsurprising to observe a limited set of engineering opportunities for neuregulin under the criterion of retaining wild-type HER4 binding and reducing HER3 binding. Many of the residues had previously been found to be integral to preserving ligand activity. The first 50–amino-acid region (Fig. 3A) was found to be the crucial core sequence of neuregulin, and truncation beyond Cys45 resulted in loss of activity^16^. Beyond the six cysteine residues that form three disulfide bonds for structural support, residues such as Gly18, Tyr/Phe40, Gly42, and Arg44 are implicated as structurally important because they are conserved across all EGF-like domains^20^. Furthermore, the β-turns between disulfide bonds (Val15-Glu19 and Gly42-Arg44) were found to be strongly affected during alanine scanning for both HER4 and HER3 binding, suggesting that these regions are important for the structural integrity of the ligand-receptor engagement. Although previous alanine scanning found that certain mutations, such as His2, Leu3 had a more pronounced effect in disrupting HER3 binding than HER4 binding, the effects were only mild when the mutants were converted to soluble protein from phage^21^. Some of the identified positions (His2, Pro29, Arg31) that mildly reduced HER3 binding while retaining HER4 binding were also found in the alanine scanning.

**Figure 4:**
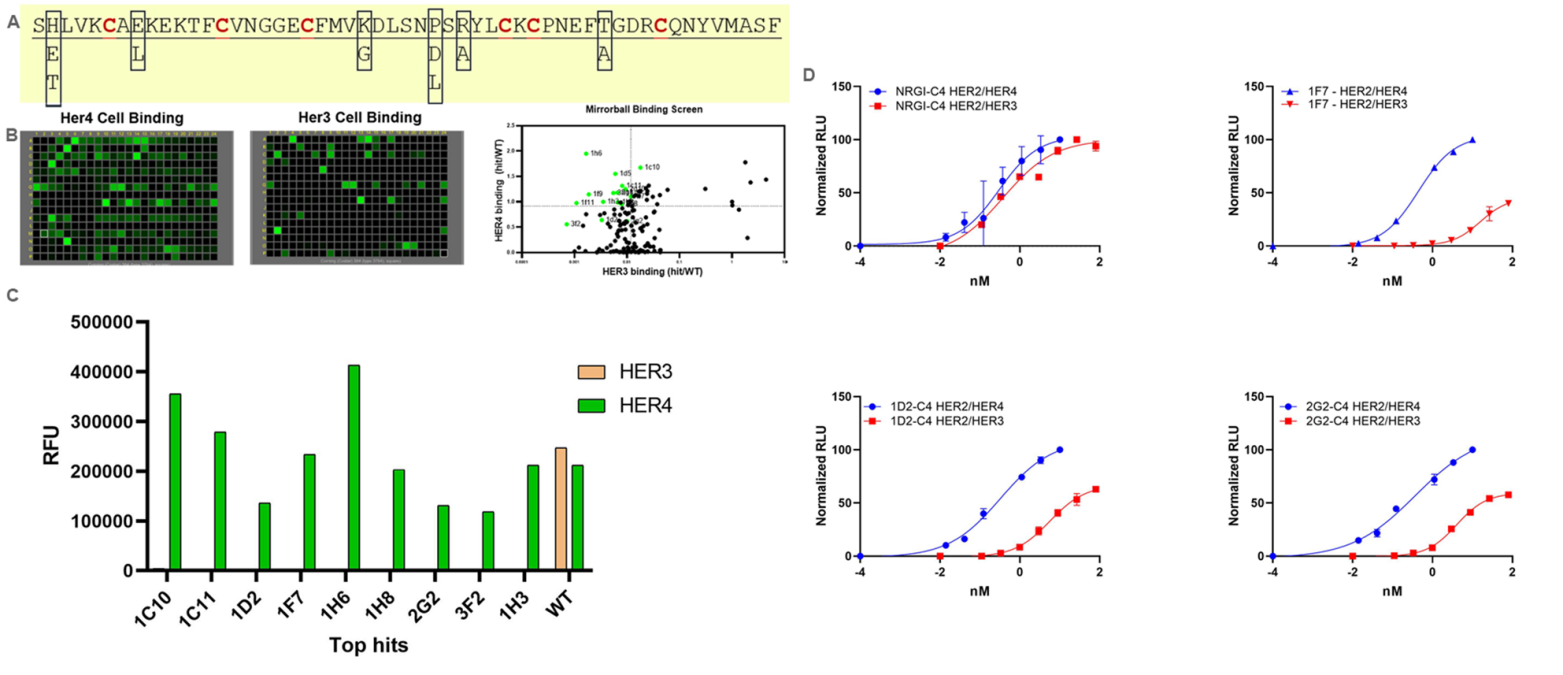
Combinatorial library construction and screening output. (**A**) A combinatorial mutagenesis library was designed to wobble all the indicated residues and their corresponding mutations from the primary screen. (**B**) A significant difference in binding signals for HER4- and HER3-overexpressing HEK293 cells was observed and quantified for clone selection at the upper left quadrant of the binding differential plot, with a variant-to-parental binding signal ratio of > 1.0 for HER4 (y-axis) and a variant/parental binding signal ratio of < 0.01 (x-axis). Cellista software version 4.2.5.0.69208 (TTP Labtech, (https://www.sptlabtech.com/) was used to create the images. (**C**) The selected hits (green) showed negligible binding to HER3. (**D**) Top clones were tested in dual-expressing HER2/HER4 and HER2/HER3 luciferase reporter cell lines. Though the effects were not as dramatic, due to the baseline expression of HER4 in the HER2/HER3 cell line, a significant increase in selective intracellular signaling activity was observed (see Table 2). *RLU* relative luminescence units, *WT* wild type.

Following the first round of saturation scanning mutagenesis library screening, we constructed a combinatorial library with all the beneficial positions simultaneously wobbled between the parental clone and the mutations (Fig. 4A). The combination clones were again expressed in high-throughput mammalian culture and the supernatants were screened separate HER4- and HER3-expressing cell binding. In this round of the binding screen, a more dramatic difference between HER4 and HER3 binding was manifested from the combination hits (Fig. 4B,C). To confirm that receptor binding selectivity conferred selective intracellular signaling activity, we generated a HER2/HER3-overexpressing luciferase reporter gene cell line that, had only low level HER4 expression from the parental HEK293 line itself (data not shown) to compare with our HER2/HER4 luciferase cell line. The top clones were probed in these cell-based HER2/HER4 and HER2/HER3 dual-expressing luciferase reporter gene assays. Whereas the parental NRG1 ligand fusion showed comparable luciferase activation for both cell lines, the selected combination hits 1F7, 1D2, and 2G2 showed selective agonistic activities for HER4-mediated activation (Fig. 4D). Sequencing revealed that a small set of mutations, including His2, Lys24, and P29, were enriched in the top hits. The EC_50_ values from these assays of the top variants are shown in Table 2.

**Table 2:** HER4-biased cell-based reporter activity in top combinatorial library hits.

Clone name	EC_50_ (nM)		Ratio	Positions
	HER2/HER4 Luc	HER2/HER3 Luc		
NRG1	0.3	0.4	1	Wild type
H2T	0.5	0.7	2	H2T
2G2	0.4	4.0	11	H2E, K24E, P29H
1D2/1C11/1H8	0.3	5.6	17	H2E, K24G
1F7	0.4	15.7	36	H2E, K24G, P29H

*Luc* luciferase.

### Retention of HER4 selectivity and increased serum half-life by ALM

**Figure 5:**
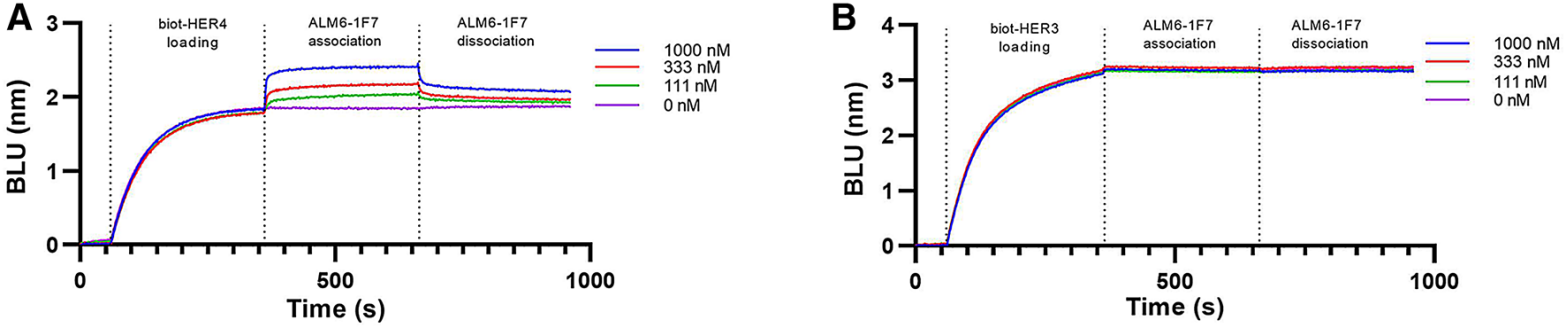
HER4-specific antibody agonists. The top NRG1 selective variant, 1F7, was introduced into the ALM6 scaffold and subsequently expressed and purified. Biolayer interferometry analysis showed HER4 binding (**A**) but no HER3 binding (**B**) at concentrations of up to 1,000 nM. *BLU* biolayer interferometry units.

Scanning mutagenesis and combinatorial selection with the neuregulin-MFc fusion platform proved to be a successful approach to the discovery of HER4-selective neuregulin variants. We set out to determine whether our strategy could be used to extend the potential of engineered ligand agonists to the antibody ligand mimetics by conferring beneficial antibody-like properties such as receptor specificity and prolonged serum half-life. To that end, we evaluated whether HER4 selectivity would be retained for these variants if they were incorporated into the ALM6 scaffold. Using a silent mutation to create BamHI restriction sites in the G4S linkers, we generated a modular version of ALM6 for a simplified plug-in construction of engineered ligand variants 1F7, 2G2, and 1D2. We compared the binding profiles of these variants to biotinylated HER4 or HER3 captured on streptavidin biosensors. The binding results showed that selectivity in the monomeric fusion proteins was indeed transferrable into the ALM IgG format (Fig. 5).

**Figure 6:**
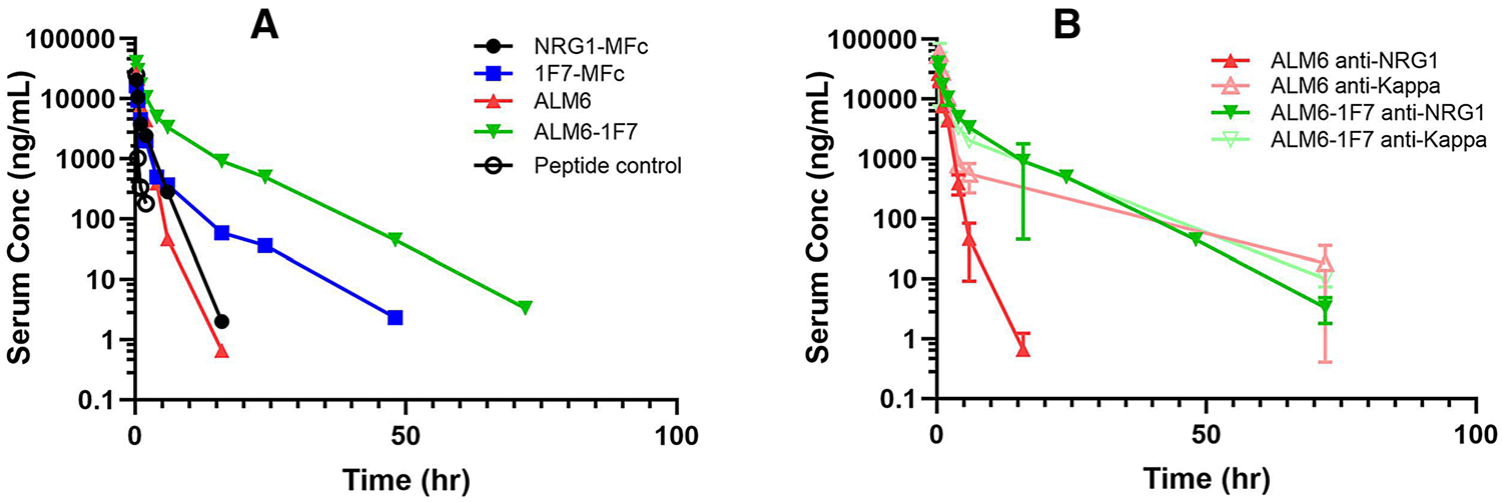
In vivo PK profiles of neuregulin fusion proteins. (**A**) hFcRn transgenic mice serum clearance curves are plotted for parental NRG1-MFc (black), 1F7-MFc (blue), parental antibody ligand mimetic ALM6 (red), and ALM6-1F7 (green), based on concurrent anti-NRG1 and Fc domain binding. Both monomeric Fc fusion and antibody ligand mimetic resulted in extended the serum circulation, compared to a peptide control (black circle). The HER4 selectivity engineered variant 1F7 acquired significant extension of serum half-life, with the lowest clearance rate from ALM6-1F7 (Table 3). (**B**) ALM6 and ALM6-1F7 serum samples were further analyzed by Fc domain and C-kappa light chain detection and compared with anti-NRG1 detection. Although both methods showed similar PK profiles for ALM6-1F7, the level of intact ALM6 molecules was lower than that of the antibody scaffold. Conc = concentration.

Like many peptide ligands, the neuregulin peptide has a very fast clearance (half-life in human around 30 min), which restricts its clinical dosing regimen to continuous infusion^9^. This well-known challenge for therapeutic peptide pharmacokinetics (PK) is related to multiple factors, including fast renal clearance due to smaller molecular size, lack of FcRn recycling, and proteolytic degradation^28^. We postulated that incorporating the neuregulin peptide into an ALM scaffold would significantly improve molecular size, FcRn recycling, and possible resistance to exopeptidase degradation. We carried out in vivo PK studies in huFcRn transgenic mice with the top selective mutant, 1F7, both as an MFc fusion protein and an ALM and compared them with their respective wild-type counterparts, NRG1-MFc and ALM6 (Fig. 6A). As expected, all the fusion protein samples extended the neuregulin serum half-life. ALM6 demonstrated a PK profile that was similar to that of NRG1-MFc, both showing some noticeable improvement from a peptide control without FcRn-mediated serum recycling. However, these molecules still displayed faster clearance and shorter half-life (Table 3) than a typical IgG (about 18 h in the huFcRn transgenic mouse model)^22^. This suggested that beyond the molecular size increase from NRG1-MFc (37 kDa) to ALM6 (140 kDa), other factors, such as protease degradation and tissue uptake, could play a significant role in protein clearance. On the other hand, both 1F7-MFc and ALM6-1F7 showed further improvement in serum concentrations, achieving almost fivefold improvement in terminal half-life compared to NRG1-MFc or ALM6. More notably, ALM6-1F7 exhibited the highest bioavailability (AUC data in Table 3) and the slowest clearance (CL in Table 3). Further probing of the PK differences between ALM6 and ALM6-1F7, using light-chain detection instead of NRG1 detection, revealed that the antibody scaffold of ALM6 persisted longer in serum, even though the CDR-embedded ligand may have been degraded (Fig. 6B). These data demonstrated that the combination of sequence optimization and increased molecular size produced the most pharmacokinetic benefit on the agonist molecule.

**Table 3:** In vivo mouse PK analysis of MFc fusion proteins and ALMs*.

Construct	Dose	C_max_	AUC_INF_	CL	t_1/2_
	(mg/kg)	(µg/mL)	(h*µg/mL)	(mL/h/kg)	(h)
NRG1-MFc	5	21	17	288	1.4
1F7-MFc	5	17	17	292	6.6
ALM6	5	27	26	194	1.4
ALM6-1F7	5	41	91	55	6.6

*PK parameters were determined by non-compartmental analysis with model 201. AUC_INF_ = area under the concentration–time curve; *CL* clearance; C_max_ = peak concentration; t_1/2_ = terminal half-life.

## Discussion

Antibody agonists are a highly desired biologic drug format due to their potential ability to surpass natural ligands by conveying additional benefits such as optimized target selectivity, enhanced activity, and prolonged half-life. Given the structural complexity of many natural ligands, it is often beyond the reach of display libraries or immune repertoires to produce antibodies with similar agonistic activities. In this work, we chose to synergize rational design and protein engineering approaches to generate antibody agonists that could selectively activate the HER4 signaling pathway, with the potential to advance cardiac regenerative treatment^3–6^. Structural insight into neuregulin bound to HER4 reveals the extensive nature of the ligand-receptor interaction and offers an intriguing opportunity for agonistic antibody discovery based on the native ligand structure. The ideal antibody agonist should possess three key design features: First, it should retain similar binding interactions between neuregulin ligand and its receptor. Second, unlike neuregulin, it should be a selective agonist of HER4, with minimal cross-activity with HER3. Third, it should offer a prolonged serum half-life, even approaching that of an IgG, in contrast to the fast clearance profiles of the natural ligand. These properties necessitated novel platform builds in conjunction with a high-throughput protein engineering workflow.

The discovery of the ultra-long CDR3H region of bovine antibody BLV1H12 has motivated several designs of agonistic antibodies to incorporate cytokines and growth factors, using the hallmark bovine stalk motif to extend the agonist structures^23–25,29^. It has been hypothesized that a rigid stalk is important to the proper folding of this unique ultralong CDR3H antibody family. As a result, both the bovine β-stranded stalk structure and a coiled-coil stalk structure have been explored to present CDR-agonist fusion proteins^30^. In our ALM designs, we envisioned a more simplified scaffolding design with a lower risk for immunogenicity, using flexible G4S linkers instead of rigid stalk-like structures to enclose the agonist sequence in the CDR3 region. We found that optimization of the linker length was paramount to generating optimal agonistic activity (Fig. 1). ALM6, which contains two G4S linkers on either side of the neuregulin sequence, produced the most optimal native ligand–like binding affinity and more potent cell-based activities. It exhibited a reasonable expression yield, in the range of 40–50 mg/L, comparable to that of previously reported yields of stalk-extended trastuzumab–CDR3H–human erythropoietin^25^. The homology structure of ALM6 illustrates that the linkers were sufficiently long to extend the agonist structure for receptor binding, with flexibility to accommodate the distance between the two termini of neuregulin (Fig. 1C). Furthermore, we improved the vector construction of ALM6 for ready plug-in of other neuregulin variants, making it a more versatile ALM platform.

An important feature of an antibody agonist is its specificity and selectivity for the target. Although a neuregulin-based peptide has been shown to have demonstrable safety profiles in preclinical models and clinical trials^31,32^, its ambivalence toward HER3 offers an opportunity to optimize its selectivity to avoid any undesired HER3 activation or off-target sink^18,19^. Prior parallel studies to probe HER3 and HER4 receptor specificity using both alanine scanning and block mutagenesis phage libraries had been conducted. It was found that binding determinants for neuregulin on both receptors are very similar, and enhanced binding variants for HER3 also commuted similar enhancements for HER4^20,21^. The cooperativity and avidity from Fc fusion proteins with dimerization of the ErbB receptors have been found to pose a challenge for binding comparisons^21^. To drive a successful ligand selectivity engineering campaign, we used a tailored MFc fusion platform to deliver a high-quality, high-throughput library selection strategy. We adopted a well-characterized MFc to build a monovalent NRG1-MFc fusion protein format for homogeneous mammalian expression to minimize false signals from protein avidity or aggregation^22^. We also needed to evaluate a comprehensive set of mutations to maximize the chances of discovering Her4 selective neuregulin variants. This MFc fusion protein served as the template for saturated scanning mutagenesis libraries to systematically survey every possible single mutation for each residue position. These point mutations offered the fine-tuning necessary to support synergistic combinations of mutations to direct protein selectivity engineering. The library diversity was easily manageable for primary and combinatorial library construction and expression. Our high-throughput cell-binding screening method using mammalian supernatants generated a good assay window for distinguishing binding to the native forms of cell surface HER3 and HER4 receptors. After the first round of saturated scanning mutagenesis, we expectedly observed very mild (two- to threefold) reduction of HER3 binding with retention of HER4 binding (Fig. 3C). Through combinatorial mutagenesis, we were able to synergize point mutations with only slight reductions of HER3 binding, to generate neuregulin variants with much more pronounced binding and activity differentiation (Fig. 4). More importantly, the selectivity was retained in the ALM agonist antibodies. Considering the high degree of structural homology between HER4 and HER3 and the challenges for identifying differentiated binders, these single and combinatorial mutations could provide further structural insight into the key epitopes that drive receptor selectivity.

**Figure 7:**
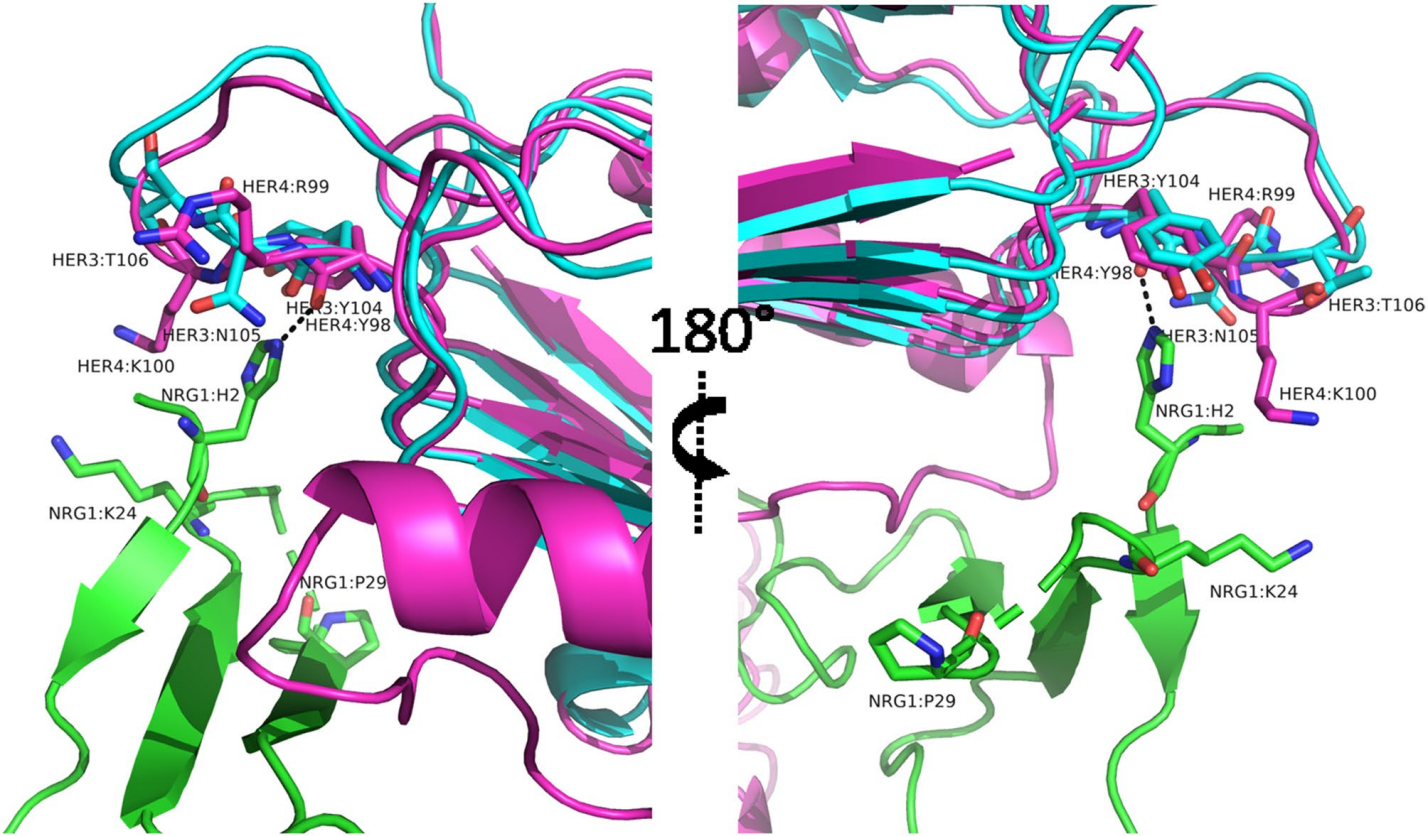
Structural view of mutations that impact HER4 selectivity. Based on co-crystal structure of NRG1 and HER4 (PDB ID: 3U7U), NRG1, colored green, is seen here to interact with the receptor HER4 (colored magenta), with HER3 (colored cyan) structure (PDB ID: 4LEO) superimposed with HER4. The three NRG1 residues (H2, K24, P29) mutated in the 1F7 clone are indicated. The histidine side chain forms a direct contact with HER4 via hydrogen bonding with the carbonyl oxygen in HER4:Y98, a conserved residue in HER3 (Y104). Two positively charged residues in HER4, R99 and K100, corresponding to N105 and T106 in HER3, are nearby and indicated. In the 1F7 clone (with mutations H2E, K24G, P29H), it is likely that the electrostatic repulsion between NRG1:K24 and HER4:K100 is removed, and reducing the conformational rigidity with the P29H mutation, the H2E mutation likely has an opportunity to fortify its interaction with HER4:R99 and K100 and maintain HER4 binding, while the same ligand mutations causes significantly reduced HER3 binding with its corresponding nonpolar residues N105 and T106. The **PyMOL** Molecular Graphics System, Version 2.4.0, Schrödinger, LLC (https://pymol.org) was used to create the structural view images.

Neuregulin selectivity engineering offered detailed biochemical and structural insight into the interaction between neuregulin and its cognate receptors HER4 and HER3. Given the challenges of generating biased HER3 or HER4 agonists, these HER4-selective neuregulin variants will be significant in dissecting the specific signaling pathways by each ErbB receptor subtype^20^. In the absence of a ligand-bound crystal structure of HER3, we superposed domain 1 from the unliganded HER3 structure with the neuregulin-bound HER4 crystal structure (Fig. 7)^14,33^. Remarkably, the mutations in the top clone, 1F7 (His2 → Glu, Lys24 → Gly, and Pro29 → His), were found to be juxtaposed in structural proximity at the same end of the three-stranded β sheet. The nearby strand-connecting loops were missing in the crystal structure, suggesting that this region is highly flexible and that it would have been difficult to achieve the selectivity engineering results only from structure-based rational design. The histidine side chain appeared to be the only one of the three residues that made direct contact with the receptor interface through hydrogen bonding with the carbonyl oxygen of the HER4 residue Tyr98. Although each individual mutation in clone 1F7 had only less than a two-fold change in receptor selectivity, the dramatic synergy from the combined mutations suggests that positions 24 and 29 can cooperatively facilitate direct engagement of position 2 with the receptor. The structural superposition of neuregulin-bound HER4 superimposed with the HER3 domain 1 offers some support for this hypothesis. On the receptor side, proximal to Tyr98, there is a difference in residue composition between HER4 and HER3. In HER4, there are two positively charged residues, Arg99 and Lys100, whereas in HER3 the residues are Asn99 and Thr100. By removing electrostatic repulsion at Lys24 in the ligand from HER4 and eliminating the conformational rigidity at Pro29, the His2 → Glu mutation could reach in further and sustain HER4 receptor binding, whereas the same mutations severely disturbed HER3 binding. Future progress to solve the neuregulin and HER3 binding structure will help illuminate the structural mechanism underlying the receptor selectivity.

Beyond retaining the selectivity from the HER4-selective neuregulin variants once they were built into the agonist antibody scaffold, we also demonstrated the PK benefits of these antibody agonists. Although the parental neuregulin ALM molecule (ALM6) improved the circulation half-life of the ligand from minutes to a few hours, the still relatively short half-life highlighted the need to address additional factors, including proteolytic liability and off-target elimination of the fusion protein. When we analyzed the serum concentration of the ALM molecules with an anti-kappa light chain detection instead of anti-neuregulin detection, we observed a longer persistence of the antibody scaffold, suggesting that the proteolysis of the neuregulin sequence in ALM6 was a contributing factor to the shorter half-life of the ligand. With the top selectivity hit ALM6-1F7, we observed greater increase in serum circulation compared with the parental ALM6 and 1F7-MFc. The increased molecular size above the renal clearance cutoff, coupled with possible proteolytic-resistant mutations in the HER4-selective antibody agonist variant, likely contributed to the improved PK. Future comprehensive profiling for proteolytic resistant variants from the mutagenesis library output, as well as developability assessment, may drive the most optimal PK improvement.

By combining the ALM scaffold design with MFc-fusion protein engineering, we were able to tailor-design agonist antibodies with enhanced receptor selectivity while capitalizing on the potential to greatly improve the PK, stability, and downstream developability profiles. This type of protein design and engineering workflow holds promise to further expand biological interrogation and drug targeting for other cardiovascular and metabolic pathways and beyond.

## Materials and Methods

### Structural modeling and analysis

Structural analysis and modeling were performed with the MOE software program (Chemical Computing Group, Montreal, Quebec, Canada) and the PyMol program (Version 2.0, Schrödinger, LLC). The following crystal structures from the Research Collaboratory for Structural Bioinformatics (RCSB) database were used: HER4 bound to neuregulin (PDB: 3U7U), unbound HER4 (PDB: 2AHX, 3U9U), unbound HER3 (PDB: 4LEO), b12 IgG1 (PDB: 1HZH), and monomeric Fc (PDB: 5HVW).

### Neuregulin-MFC fusion proteins and ALM expression and purification

All chemicals were of analytical grade. Oligonucleotides and NRG1b gene fragments were synthesized by Eurofins MWG Operon (Louisville, KY). For the construction of neuregulin-MFC fusion proteins, plasmids encoding the EGF domain of NRG1b fused with a previously well-characterized MFc (C4) were generated, using the In-Fusion HD cloning kit (Takara Bio, Mountain View, CA), into an in-house mammalian expression vector^22^. For ALM constructions, human IgG1 b12, with well-known crystal structure (PDB: 1HZH) as well as non–cross-reactivity toward any human proteins, was chosen as the candidate backbone^26^. B12 variable domain fragments containing neuregulin peptides with various linkers were incorporated into either the antibody variable heavy-chain region of CDR3 or the variable light-chain region of CDR1. Fusion protein or antibody variants were transiently transfected into the human embryonic kidney cell line HEK293FT, using 293Fectin transfection reagent (Life Technologies, Carlsbad, CA). Cells were grown in FreeStyle 293-F Expression Medium (Life Technologies). The expression levels of all antibody and fusion variants were evaluated with an Octet QK 384 instrument (ForteBio, Menlo Park, CA), using protein A sensors. The expressed antibodies were purified from cell supernatant by affinity chromatography, using a HiTrap Protein A column (GE Healthcare Life Sciences, Marlborough, MA). Monomer content for all the antibodies and fusion proteins was determined by analytical SEC-MALS.

### Ligand mutagenesis library construction and screening

Site-directed mutagenesis was performed with the Quikchange Lightning Multi site-directed mutagenesis kit (catalog number 210514; Agilent Technologies, Santa Clara, CA). Degenerate NNS primers were synthesized by Eurofins Genomics (Louisville, KY) for each position in the neuregulin peptide, excluding the six cysteine residues to preserve the disulfide bonds. Libraries of 12–18 positions each were combined for a total of three individual libraries. Colonies were picked in 96-well format and grown in 2-YT broth overnight. NucleoSpin 96 plasmid core kits (catalog number 740625; Macherey–Nagel, Bethlehem, PA) were used to prepare DNA for sequencing and transient transfection. Protein expressed in the supernatant of 293 cells was then used for screening. After the primary library screening, site-directed mutagenesis was also used to generate a combinatorial library to simultaneously wobble the top point mutations with their parental residues.

### Binding ELISAs and affinity measurements

Measurement of binding of the fusion proteins or ALMs to in-house purified recombinant human HER4 or HER3 was carried out by biolayer interferometry on an Octet 384 instrument (ForteBio). Biotinylated HER4 or HER3 at 1 μg/mL in phosphate-buffered saline (PBS) (pH 7.4) with 3 mg/mL bovine serum albumin, 0.05% (vol/vol), Tween 20 (1 × Kinetics Buffer; ForteBio) were captured on streptavidin biosensors (ForteBio). The loaded biosensors were washed with assay buffer to remove any unbound protein, after which association and dissociation measurements were conducted with serial dilutions of the different variants. Affinity calculations were based on steady-state association curve fitting analysis. Processing of data and measurements of dissociation rate constants were conducted with Octet Software, version 7.1.

### Generation of luciferase reporter cell lines

HER2-expressing HEK293 cell line with high HER2 expression had been generated previously and grown in DMEM + 10%FBS medium. Both these cell lines were transduced with lentivirus containing the full length HER4 gene or HER3 gene, along with the luciferase gene. Three days after transduction, the cells were put under selection with 10 μg/ml blasticidin for HER4 selection, 1 μg/ml puromycin for HER3 selection, and 100 μg/ml hygromycin B for luciferase selection, for seven days. After selection cells were confirmed to express HER4 or HER3, with luciferase.

### Cell-based receptor binding assays

Mammalian supernatants from NRG-1 libraries were subjected to high-throughput screening with a stable HEK293 cell line that expressed HER3 or HERr4, using a no-wash cell-binding assay. Each cell line was plated in an individual 384-well plate for comparison at 4 × 10^5^ cells per well in 20 μL of Freestyle 293 expression medium (Thermo Fisher Scientific, Waltham, MA). Supernatant from the libraries was added to each well at 10 μL per well. Goat antihuman Alexa Fluor 647 was then added at a final concentration of 600 ng/mL. Assay plates were then incubated in the dark for 4–6 h and read on the Mirrorball System (SPT Labtech, Hertfordshire, UK). Data were analyzed by comparing the ratio of HER3 to HER4 signal intensity.

Flow cytometry was used for confirmation. The same stable cell lines as described above were blocked with PBS + 1% fetal bovine serum for 1 h, washed, and incubated at 4 °C with 1 and 10 μg/mL purified NRG-1 mutants for 1 h. Cells were then stained with goat antihuman Alexa Fluor 647 (1 μg/mL), washed, and read on a flow cytometer (BD FACSAria Fusion Special Order System; BD Biosciences, San Jose, CA). Data were analyzed with FlowJo software (version 10.4.1; FlowJo, Ashland, OR).

### Cell-based HER4 and HER3 signaling assays (luciferase and iPSC-derived cardiomyocytes)

HER2-HER3 and HER2-HER4 cells were cultured at 37 °C, 50% CO_2_ to about 70–90% confluency. The cells were resuspended in serum-free Dulbecco modified Eagle medium at a final concentration of 2 × 10^4^ cells per mL per well of white, flat-bottomed, 96-well plates. The ligands and antibodies were added to the cells and incubated for 6 and 24 h, respectively. BrightGlo assay reagent (Promega, Madison, WI) was added to the cells. Luminescence signals were measured with an EnVision reader (PerkinElmer, Waltham, MA) according to the manufacturer’s instructions.

### In vivo PK assays

A quantitative ELISA was used to monitor the serum concentrations of the tested antibodies. For detection of NRG1, 1F7, AM6, and AM6-1F7, 96-well plates were coated with 10 μg/mL goat anti-mouse IgG1-Fc (Jackson ImmunoResearch, West Grove, PA) and incubated overnight at 4 °C. The plates were blocked with 3% bovine serum albumin in PBS-Tween for 1 h and incubated with 8 μg/mL mouse anti-NRG1 antibody (Thermo Fisher Scientific) for 1 h at room temperature. The plates were then incubated with the diluted serum samples at different time points. Goat antihuman Fc-specific horseradish peroxidase–conjugated antibody (dilution 1:10,000; Jackson ImmunoResearch) was used for detection. Absorbance at 450 nm was measured after development with 3,3′,5,5′-tetramethylbenzidine substrate (Kirkegaard & Perry Laboratories, Gaithersburg, MD) according to the manufacturer’s directions. Standard curves were generated for each antibody variant. The linear portions of standard curves generated in Prism (version 6; GraphPad Software, La Jolla, CA) were then used to quantify NRG1, 1F7, AM6, and AM6-1F7 fusion proteins in the serum samples. Non-compartmental PK data analysis was performed using Phoenix 64 WinNonlin 8.1 (Pharsight, Mountain View, CA), as previously described^22^. Briefly, the maximum observed peak plasma concentration (C_max_) was determined by inspection of the observed data using WinNonlin. The terminal elimination half-life (t_½_) was determined using the equation ln(2)/z, where z is the slope of the terminal portion of the natural-log concentration–time curve, determined by linear regression of at least the last 3 time points. The systemic exposure was determined by calculating the area under the curve for the plasma concentration versus time graph (AUC_last_) from the start of dosing to the time of last measurable concentration using the linear/log trapezoidal rule. Area under the curve for the plasma-concentration vs. time graph from time 0 to infinity (AUC_∞_) was calculated as: AUC_last_ + C_last_/λz, where C_last_ is the last quantifiable concentration. Clearance (CL) was calculated by dose/AUC_∞_, and steady state volume of distribution (V_ss_) was calculated as: (AUMC_∞_ × CL)/AUC_∞_, where AUMC_∞_ is the area under the curve from the first moment extrapolated to infinity.

## Data Availability

All data needed to evaluate the conclusions in the paper are present in the paper. Additional data related to this paper may be requested from the authors.
